# Metal uptake in sweet peppers cultivated in soils contaminated by artisanal gold mining: implications for food safety

**DOI:** 10.1007/s10653-026-03077-z

**Published:** 2026-03-09

**Authors:** Elvia Valeria Durante-Yánez, Iván David Urango-Cárdenas, Germán Holland Enamorado-Montes, Marisol Laza-Durante, Enrique Combatt Caballero, José Marrugo-Negrete, Roberth Paternina-Uribe, Sergi Díez

**Affiliations:** 1 Faculty of Basic Sciences, Department of Chemistry, Water Research Group, Applied Chemistry and Environmental, Universidad de Córdoba, Monteria, Colombia; 2 Faculty of Engineering, Universidad de Córdoba, Monteria, Colombia; 3 Environmental Chemistry Department, Institute of Environmental Assessment and Water Research, IDAEA-CSIC, 08034 Barcelona, Spain

**Keywords:** Mercury, Lead, Arsenic, *Capsicum annuum*, Bioconcentration, Translocation factor

## Abstract

Soil contamination by metals poses a significant threat to food safety and human health, particularly through the consumption of vegetables cultivated in mining-impacted areas. In Colombia, gold mining and horticultural activities frequently coexist; however, information on the uptake of mining-associated metals by edible crops and the resulting health risks remains limited. This study investigated the accumulation of mercury (Hg), lead (Pb), and arsenic (As) in sweet pepper (*Capsicum annuum*) cultivated in soils affected by gold mining and evaluated the potential human health risks. A single-factor experimental design was employed using soils collected at three distances from active mining sites (S1: 0.6 km, S2: 3 km, and S3: 20 km). Plants were grown under screen-house conditions for 143 days, during which morphometric, physiological, and chemical analyses were performed. Soil concentrations of metals decreased with increasing distance from mining activity: S1 (Hg: 22.13, Pb: 1997.02, As: 37.52 mg kg^−1^), S2 (Hg: 5.38, Pb: 186.03, As: 15.70 mg kg^−1^), and S3 (Hg: 2.05, Pb: 57.19, As: 7.90 mg kg^−1^), with generally low bioavailability (Hg and As < 1%; Pb: 2–11%). Metal accumulation occurred predominantly in roots, with limited translocation to aerial and edible tissues. Bioconcentration factor (BCF) and translocation factor (TF) were consistently below unity, indicating limited uptake and internal transfer of Hg, Pb, and As. Human health risk assessment based on Codex Alimentarius provisional tolerable weekly intake (PTWI), margin of exposure (MOE), total hazard quotient (THQ), and incremental lifetime cancer risk (ILCR) indicated low non-carcinogenic risk and negligible carcinogenic risk at distances of 3 and 20 km. Although Hg concentrations exceeded the limits established by the Chinese National Food Safety Standard (GB 2762-2022), which sets a threshold of 0.1 mg kg^−1^, the overall results suggest no immediate health risk from consumption. Nevertheless, considering the persistence and bioaccumulative nature of these elements, long-term exposure risks cannot be excluded, highlighting the need for continuous monitoring of agricultural systems in mining-influenced regions.

**Supplementary Information:**

The online version contains supplementary material available at 10.1007/s10653-026-03077-z.

## Introduction

Metal and metalloid contamination presents a significant risk to both human and environmental health, with lasting impacts on ecosystems (water, air, and soil) and potential for bioaccumulation in the food chain (Caicedo-Rivas et al., 2023; Islam et al., 2025; Olivero-Verbel et al., 2015). The rising concern over metals such as mercury (Hg), lead (Pb), and arsenic (As) in soils is driven by their ability to be absorbed by food crops, posing a serious food safety risk. These toxic elements can lead to severe health issues in humans, from organ damage to cancer (Jomova et al., 2025; Marrugo-Madrid et al., 2022). The impact of these elements on plants, however, depends not only on their soil concentrations but also on their bioavailability (Kaninga et al., 2020; Zhao et al., 2024).

In Colombia, heavy metal contamination in soils is often attributed to the improper disposal of gold mining waste (Betancur-Corredor et al., 2018; MinMinas & UNODC, 2022). The Bolívar department, one of Colombia’s leading gold mining regions, reports notably high metal concentrations in its soils (Rocha-Román et al., 2018; Sistema de Información Minero Colombiano & Unidad de Planeación Minero Energético, 2025). This is concerning because the department of Bolívar is one of the country’s leading producers of vegetables, including sweet pepper (*Capsicum annuum*), with an estimated production of 9,722 tons in 2024 (Asociación Hortifrutícola de Colombia—ASOHOFRUCOL, 2024; Martínez Reina et al., 2020).

*C. annuum* is widely consumed across the globe, valued for its rich content of essential nutrients such as vitamin C, provitamin A, B vitamins, calcium, minerals, carotenoid antioxidants, capsaicinoids (capsaicin), and tocopherols (Choi et al., 2023). The health benefits of consuming *C. annuum* are significant, as these nutrients contribute to cellular protection and reduce the risk of various diseases, including cancer, diabetes, cardiovascular disorders, cataracts, Alzheimer’s, and Parkinson’s disease (Cortés-Estrada et al., 2020). However, like many plants, *C. annuum* can adapt to stress in metal-contaminated soils, though its growth, development, and productivity are often hindered due to phytotoxicity (Hu et al., 2017; Hussain et al., 2022). Research has documented Hg, Pb, and As levels in the fruits of different *C. annuum* species grown in mining regions worldwide, including Colombia (Bundschuh et al., 2012; Jiang et al., 2025; K. Luo et al., 2022; Marrugo-Negrete et al., 2016a, 2016b; Pérez Vargas et al., 2014; Zhao et al., 2024; Zhou et al., 2016). In the study area located Mina Santa Cruz (Barranco de Loba, Bolívar, Colombia), Córdoba-Tovar et al. (2025) reported concentrations of potentially toxic elements, including Hg, Pb, and As, in several plant species, including *C. annuum*. Also, Pérez-Vargas et al. (2014) reported Hg concentrations in fruits, leaves, stems, and roots of *C. annuum* grown in soils from Mina Santa Cruz. Although the values obtained could be considered low in relation to the permitted weekly intake, it is important to note that, despite not exceeding the established levels, Hg is a highly toxic element even in small amounts, according to the World Health Organization (WHO, 2024). These findings raise serious concerns about the potential adverse effects of these metals on the health of consumers of this vegetable. Although studies of this type have been reported, there is still a void in Colombia regarding the accumulation of metals in vegetables consumed daily and how the bioavailability of these toxic elements determines their actual risk to human health. In this context, the lack of comprehensive studies limits the formulation of evidence-based agricultural and health policies and delays the implementation of management practices that reduce the population exposure.

Therefore, the objective of this research was to evaluate the accumulation of Hg, Pb, and As in *C. annuum* grown in soils contaminated by gold mining activities in San Martín de Loba, Bolívar. For this purpose, the concentrations of Hg, Pb, and As were determined in soils and across different plant organs. In addition, bioconcentration factor (BCF) and translocation factor (TF) were calculated to evaluate contaminant accumulation and root‑to‑shoot transfer efficiency in the plant. Plant tolerance and growth were evaluated through morphological and physiological traits. Likewise, the weekly intake of each metal was estimated to determine the risk associated with the consumption of *C. annuum* grown in contaminated soils, considering their bioavailability. The results of this study constitute relevant scientific evidence to promote food safety and health protection for communities exposed to the consumption of vegetables grown in areas impacted by gold mining.

## Materials and methods

### Description of the soils

The municipality of San Martín de Loba, in Bolívar Department, is one of the largest gold mining areas in Colombia. In addition to mining, residents also engage in agriculture and cultivate vegetables in small orchards. However, these areas are affected by the improper disposal of mining waste, leading to significant contamination with metals and metalloids. (Olivero-Verbel et al., 2015; Pérez Vargas et al., 2015).

**Fig. 1:**
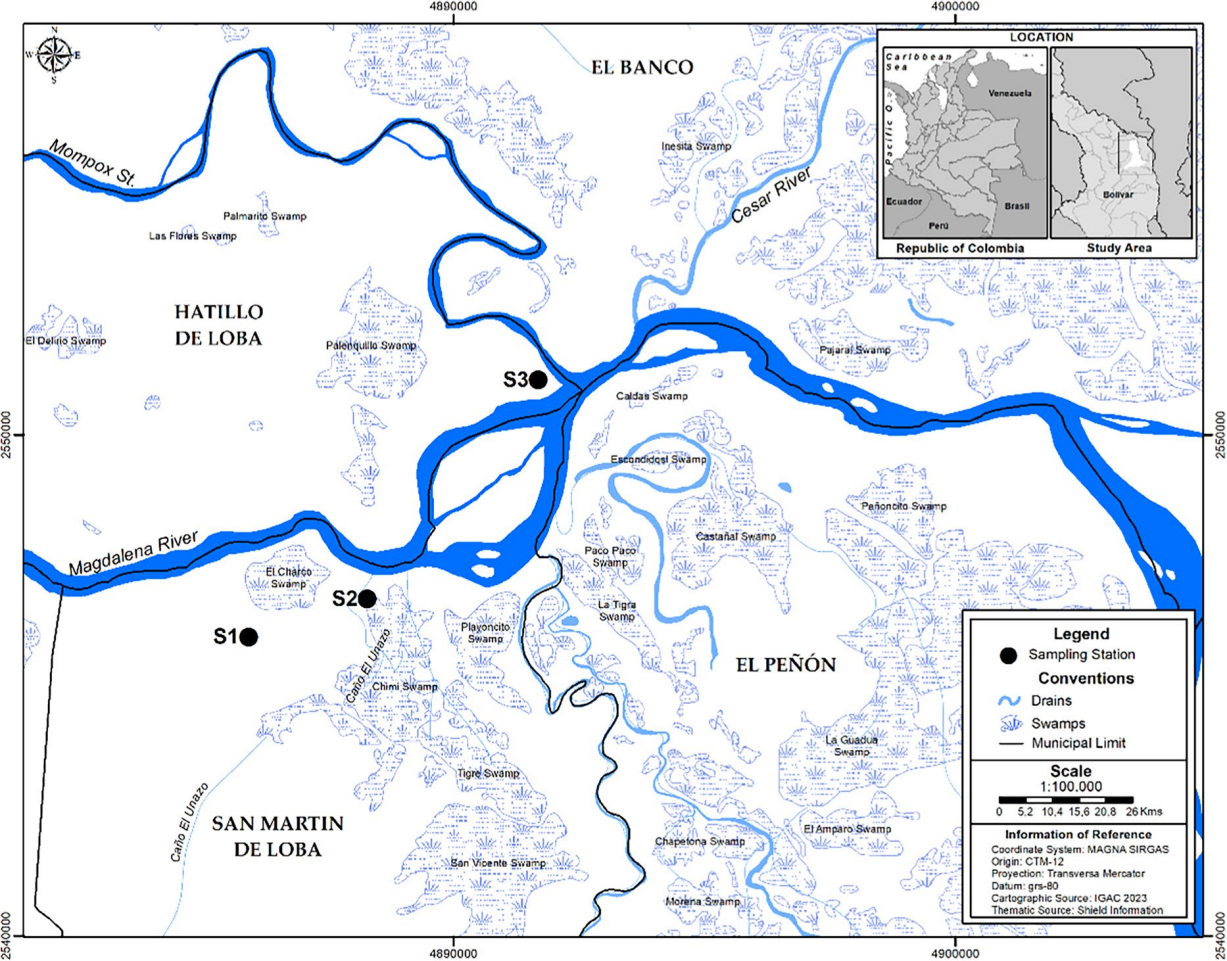
Soil sampling stations of Bolívar Department, Colombia

Therefore, three sites were selected for soil collection, as shown in Fig. 1. To examine the effect of soil proximity to gold mining sites, we collected soil samples from three distinct distance levels: S1: approximately 0.6 km from a mine (8° 56′ 21.9″ N 74° 02′ 17.3″ W), S2: agricultural soils located 3 km away from mining activities (8° 56′ 46.8″ N 74° 00′ 59.9″ W), and S3: minimally intervened soils situated 10 km from mining areas (8° 59′ 09.4″ N 73°59′08.5″ W). At each site, soil samples were collected from an area of 10 m^2^ at a depth of 30 cm (Smolińska & Cedzyńska, 2007). Within this area, a composite soil sample (90 kg) was prepared by randomly collecting three subsamples of 30 kg each. Soil samples were transported to the Toxicology and Environmental Management Laboratory of the University of Córdoba (8° 56′ 15.9″ N, 74° 02′ 39.1″ W), air-dried at ~ 35 °C, homogenized, and sieved (< 2 mm) (J. Luo et al., 2017).

### Physicochemical analysis and environmental risk of soils

After drying and sieving process, soil samples were collected for the purpose of determining physicochemical parameters and analyzing Hg, Pb, and As concentrations, as well as their bioavailability. Soil physicochemical properties were analyzed using standardized methods. Soil pH was measured in a 1:1 soil-to-water suspension with the potentiometric method (NTC 5264; ICONTEC, 2008). Organic matter (OM) content was determined by the Walkley–Black method (NTC 5403; ICONTEC, 2013). Available sulfur (S) and phosphorus (P) were quantified using the 0.008 M calcium monophosphate and Bray II methods, respectively. The effective cation exchange capacity (ECeC) was estimated as the sum of Ca, Mg, K, and Na, determined by extraction with 1.0 M ammonium acetate at pH 7.0 (IGAC, 2006). Finally, soil texture was analyzed using the Bouyoucos hydrometer method (IGAC, 2006).

The concentration of Hg was analyzed in accordance with the EPA 7473 method, employing a direct mercury analyzer DMA-80 Tricell (Milestone, Sorisole, Italy) (USEPA, 2007b). Approximately 0.05 g of homogenized sample was directly weighed into the analyzer for thermal decomposition, amalgamation, and atomic absorption detection. For the concentration of Pb and As, the samples were subjected to microwave-assisted acid digestion using a Ethos One digestion system (Milestone srl, Sorisole, Italy), with nitric acid and hydrogen peroxide (analytical grade, Merck, Germany) following the EPA 3051A method (USEPA, 2007a). A total of 0.3 g of dried and homogenized sample was digested with 10 mL of an acid mixture consisting of HNO_3_:H_2_O_2_ (8:2, v/v). After digestion, the extracts were diluted to a final volume of 50 mL with ultrapure water (Type I). Pb quantification was performed by flame atomic absorption spectroscopy reading using a Thermo Scientific ICE 3500 atomic absorption spectrometer (iCE 3500, Thermo Fisher Scientific, Waltham, MA, USA). For As analysis, after microwave-assisted acid digestion (EPA 3051A), samples were added HCl, KI and ascorbic acid, followed by quantification using a Thermo Electron IC3500 atomic absorption spectrometer with a VP100 hydride generation system (Thermo Scientific, MA, USA).

Quality control for soil metal determination included the use of method blanks, triplicate analyses, and a certified reference material (CRM008-050; Resource Technology Corporation, USA) to ensure both accuracy and precision. Method blanks were processed with each digestion batch to rule out contamination, while 10% of the total samples were analyzed in triplicate, yielding relative standard deviations (RSD) below 10%. Accuracy was evaluated using the certified reference material, yielding mean recoveries of 96.8% for As, 101.4% for Pb, and 98.3% for Hg, with coefficients of variation below 5%, all within the 95% confidence limits. Limits of detection (LOD), calculated as three times the standard deviation of seven method blanks, were 0.001 mg kg^−1^ for Hg, 0.080 mg kg^−1^ for Pb, and 0.050 mg kg^−1^ for As, while limits of quantification (LOQ) were defined as ten times the standard deviation (0.003 mg kg^−1^ for Hg, 0.270 mg kg^−1^ for Pb, and 0.180 mg kg^−1^ for As). Instrument calibration was verified at the beginning of each analytical run using multi-element standards (Merck, Germany), and continuing calibration checks were performed every 10 samples.

To assess the environmental risk of soils, the Risk Assessment Code (RAC) was implemented, focusing on the first two bioavailable fractions of metals in soils according to sequential extraction methods. For Hg, the method developed by Bloom et al. (2003) is employed, defining two bioavailable fractions: F1, soluble in water (extracting HgCl_2_ and HgSO₄ using deionized water), and F2, soluble in human stomach acid (extracting HgO using a solution of 0.01 M HCl and 0.1 M CH_3_COOH).

For Pb and As, the methodology of Tessier et al. (1979) is applied, where the bioavailable phases are F1, the exchangeable fraction (extracting exchangeable metals with 1 M MgCl_2_, e.g., PbE and AsE), and F2, the carbonate fraction (extracting metals associated with carbonates using 1 M NaOAc, e.g., PbC and AsC). The RAC value is then calculated using the following equation (Cheng et al., 2018). All reagents used in this study were of analytical grade and supplied by Merck (Germany).

1 $$ RAC = F_{{1_{ } }} + F_{2} $$

where F1 and F2 correspond to the percentage of metal (Hg, Pb and As) extracted in the first and second fractions of the methods, respectively. The RAC is categorized into five levels of environmental risk: No risk (RAC < 1%); low risk (1% ≤ ACR < 10%); medium risk (11% ≤ ACR < 30%); high risk (31% ≤ RAC < 50%); very high risk (ACR ≥ 75%).

### Design and experimental development

This study employed a unifactorial design based on the distance of soil samples from gold mining activities, with three levels (0.6 km, 3 km, and 10 km) and three replicates per level, resulting in a total of nine experimental units.

The experiment was carried out under controlled conditions in a screen house of the University of Córdoba between March and May 2018. Average screen-house conditions during the trial were approximately 35 °C and 49% relative humidity. Nine pots containing the three soil types were arranged randomly and rotated every 15 days. *C. annuum* seeds (*C. annuum*) were obtained from local inhabitants within the study area. The material was subsequently authenticated by professionals from the University of Córdoba, who taxonomically verified and confirmed that all seeds corresponded to a single variety. The seeds were sown in a seedbed, and after 45 days of germination, the seedlings were transplanted into each experimental unit. Throughout the experiment (143 days), plants were irrigated to three-quarters of field capacity to prevent leaching (Marrugo-Negrete et al., 2015). At harvest, physiological variables were measured, including stem length (from base to apex, using a graduated ruler, recorded in centimeters (cm)), stem diameter (measured at the base with a vernier caliper, recorded in millimeters (mm)), number of leaves, and number of fruits (counting only fully developed structures).

At the end of the trial, the plants were harvested and rinsed with metal-free water to remove any adhering soil particles. The plants were then divided into roots, stem, leaves, and fruits, and each part was weighed separately. Additionally, post-harvest soil samples were collected from each experimental unit for subsequent analysis.

### Determination of leaf area, dry biomass, chlorophyll and carotenoids

Leaf area was calculated using the weight-to-area ratio, also known as the "punch" method, with a 2.7 cm diameter punch corresponding to an area of 5.72 cm^2^ (Pire & Valenzuela, 1995). Biomass for each plant organ (root, stem, and leaf) was determined by drying the samples in an oven at 40 °C for four days, after which they were weighed to obtain the dry matter using an Ohaus Adventure model analytical balance (Marrugo-Negrete et al., 2016a, 2016b).

Chlorophyll and carotenoid contents were measured using the Lichtenthaler method (1987). In this method, 0.5 g of chopped leaves were added to an 80% acetone solution and kept in the dark for 24 h. The chlorophyll and carotenoid concentrations were then measured by UV–Vis spectroscopy using a Perkin Elmer Lambda 11 spectrometer, following specific absorption coefficients at A663, A647, and A470. The equations used for calculating the concentrations of chlorophyll A (Eq. 2), chlorophyll B (Eq. 3), total chlorophylls (Eq. 4), and total carotenoids (Eq. 5) in the leaf pigment extracts (v/v) are as follows:

2 $$ Chlorophyll \; {A} = 12.25A_{663} - 2.79A_{647} $$

3 $$ Chlorophyll  \; {B} = 21.50A_{647} - 5.10A_{663} $$

4 $$ {{Chlorophyll}} \; {A} + {B} = 7.15{A}_{663} + 18.71{A}_{647} $$

5 $$ {{Carotenoids}} = \frac{{1000{A}_{470} - 1.82{{Chlorophyll}}{A} - 85.02{{Chlorophyll}}{B}}}{198} $$

### Determination of concentration of Hg, Pb and As in plants

The concentration of Hg, Pb and As in plants were quantified according to the EPA 7473 (for Hg) and EPA 3051A (for Pb and As) methods described in Sect. "Physicochemical analysis and environmental risk of soils". It should be noted that the samples of the plants divided into roots, stems, leaves and fruits were dried and homogenized by grinding prior to analysis (USEPA, 2007a, 2007b).

As a quality control measure, 10% of the samples were analyzed in duplicate and quantified using a calibration curve, with a determination coefficient greater than or equal to 0.9995 for all metals. Additionally, the detection limit for each metal, determined as three times the standard deviation of 10 blank measurements, was 50 µg As kg^−1^, 80 µg Pb kg^−1^, and 0.1 µg Hg kg^−1^. To verify the method’s traceability, we evaluated it by analyzing IAEA-336 certified reference material (Trace elements in lichens) from the International Atomic Energy Agency in triplicate (certified value = 0.63 ± 0.08 μg As/g; 0.117 ± 0.017 μg Pb g^−1^, 0.2 ± 0.04 μg Hg g^−1^). The recovery percentage for As (0.67 ± 0.04 μg g^−1^), Pb (0.121 ± 0.010 μg kg^−1^), and Hg (0.21 ± 0.03 μg g^−1^) in IAEA-336 was 106.3%, 103.4%, and 105%, respectively, with coefficients of variation below 5%, all within the 95% confidence limits.

### Determination of translocation and bioconcentration factors

From the concentrations of Hg, Pb, and As in the plant organs and soils, TF and BCF were calculated. The TF for metals in plants was determined by dividing the metal concentration in the leaves by the concentration in the roots (Eq. 6). The BCF was calculated as the ratio of metal concentration in the roots to the total metal concentration in the soil (Eq. 7) (Marrugo-Negrete et al., 2020; Ruiz Huerta & Armienta Hernández, 2012). The equations used are provided below:

6 $$ TF = \frac{{\left[ {Metals\;in\;leaves} \right]}}{{\left[ {metals\;in\;roots} \right]}} $$

7 $$ BCF = \frac{{\left[ {Metals\;in\;roots} \right]}}{{\left[ {Metals\;in\;soil} \right]}} $$

### Health risk assessment

Health risks from the consumption of *C. annuum* fruits were assessed considering both non-carcinogenic and carcinogenic effects, following the US EPA risk assessment framework. Non-carcinogenic risk was evaluated using the Total Hazard Quotient (THQ), expressed as:

8 $$ THQ = CDI/RfD $$

where CDI is the chronic daily intake (mg kg^−1^ BW day^−1^) and RfD is the oral reference dose (mg kg^−1^ day^−1^). RfD values were obtained from USEPA databases: Pb = 0.0035, As = 0.0003, Hg = 0.0003 (Laboni et al., 2023), and CDI was calculated as:

9 $$ CDI = \frac{EDI \times EF \times  ED}{{AT}} $$

where EF = exposure frequency (365 days year^−1^), ED = exposure duration (70 years), and AT = average exposure time (25,550 days), and the estimated daily intake (EDI, mg kg^−1^ day^−1^) was calculated the following equation (Shaheen et al., 2016):

10 $$ EDI = \frac{FIR \times C}{{BW}} $$

where FIR is the food intake rate (g/person/day), C is the metal concentration in food samples (mg kg^−1^), and BW is body weight.

In our study, EDI was calculated from the average concentration of each metal in the fruits from each harvest and the daily consumption rate of the study area. According to Pérez-Vargas et al. (2015), in the South of Bolívar, there is an average consumption of 250 g per week (35.7 g/day) of *C. annuum*. For environmental risk assessment studies, an average body weight (BW) of 70 kg is considered.

For mercury (Hg), exposure was evaluated in relation to the Provisional Tolerable Weekly Intake (PTWI) established by international health authorities (JECFA, 2011). For lead (Pb) and arsenic (As), since no PTWI values are currently established, risk characterization was conducted using the Margin of Exposure (MOE) approach, based on benchmark dose lower confidence limits (BMDL) derived from epidemiological studies. The MOE was obtained as the ratio between the BMDL and the EDI. In accordance with EFSA guidance, higher MOE values indicate a lower health concern, while lower values suggest a narrower margin of safety. Specifically, for Pb and As, which are considered genotoxic and carcinogenic, relatively small MOE values highlight the potential for concern, particularly in sensitive populations (EFSA Panel on Contaminants in the Food Chain (CONTAM), 2010).

The cumulative non-carcinogenic risk was calculated as the Hazard Index (HI):

11 $$ HI = \mathop \sum \limits_{i = 1}^{n} THQ $$

with *i* representing each heavy metal in the sample. An HI < 1 indicates negligible risk, while HI > 1 suggests potential adverse health effects and the need for intervention.

Carcinogenic risk was estimated using the Incremental Lifetime Cancer Risk (ILCR), defined as:

12 $$ ILCR = CDI \times CSF $$

where CSF is the oral cancer slope factor (mg kg^−1^ day^−1^), with values adopted from OEHHA (Pb = 0.0085; As = 1.5), and classified according to US EPA criteria: ILCR < 10^−6^ (negligible), 10^−6^–10^−4^ (acceptable), ILCR > 10^−4^ (potential concern), and ILCR > 10^−3^ (moderate risk, public health issue) (Afrin et al., 2022; Laboni et al., 2023).

### Statistical analysis

Results are presented as mean ± standard deviation. For the statistical analysis, ANOVA and Tukey’s mean comparison test were performed, at a significant level of 95% (*p* ≤ 0.05) using the statistical software Statistica 12.0 from Statsoft and the GraphPad Prism 5 Software was used to make the graphs. Tukey’s test was used when the Anova was significant.

## Results and discussion

### Physicochemical characteristics of soils

The physicochemical characteristics of the soils are presented in Table 1. The silty loam texture of S1, and the loamy texture of S2 and S3, are suitable for agricultural use. Porta et al. (1994) posit that the proportions of sand, clay, and silt provide favorable conditions for aeration, drainage, and moisture retention, thereby promoting optimal plant development.

**Table 1:** Physicochemical characteristics of the soils at increasing distances from mining activities (S1: 0.6 km; S2: 3 km; S3: 20 km)

Code	Texture	pH	OM	S	P	Al + H	CIC_e_
		1:1	%	mg kg^−1^		Cmol kg^−1^	
S1	Silty loam	4.62	1.99	444.56	13.33	2.34	12.99
S2	Loamy	4.8	2.05	553.93	22.35	1.02	15.33
S3	Loamy	4.78	1.74	152.6	21,7	0.60	9.9
After dolomitic lime application							
S1	Silty loam	6.55	1.45	437.89	11.63	0.17	13.44
S2	Loamy	6.69	1.78	494.37	21.39	–	16.42
S3	Loamy	6.70	1.39	142.08	19.89	–	10.19

The soils in sites S1 and S2, due to their initial chemical characteristics, can be classified as acid sulfate soils (SSA), since they have an acid character evidenced by the pH, high S content and high values of exchangeable aluminum (Al + H) (Combatt Cabellero et al., 2003; Gómez, 2005). The soil in S3 exhibits an acidic pH, elevated concentrations of S, and optimal levels of Al + H. (Gómez, 2005). For the three soils, the OM and CICe are in a medium range (Gómez, 2005). OM in soils has the capacity to bind with toxic metals, thereby regulating their mobility. This phenomenon is attributed to the propensity of metals to form stable complexes with organic ligands (Sofianska & Michailidis, 2015). To neutralize the acidity of the soils, since this can contribute to the mobility of metals and increase bioavailability when it is less than 4 (Khan et al., 2015), 20 g of dolomitic lime was applied with a relative power of total neutralization of 92% in each pot and, after three months of application, the pH increased to values ​​greater than 6 (Table 1), then sowing was carried out.

**Fig. 2:**
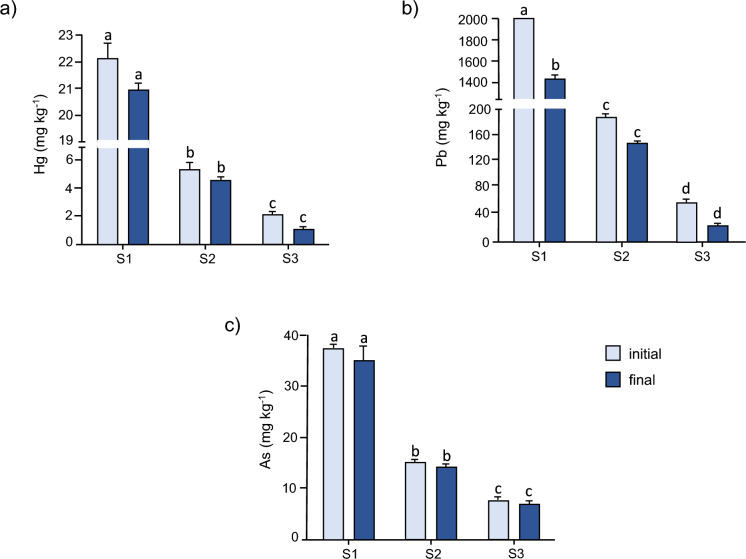
Initial and final concentrations of Hg, Pb and As. In the soils at increasing distances from mining activities (S1: 0.6 km; S2: 3 km; S3: 20 km)

The initial and final concentrations of Hg, Pb, and As (Fig. 2) in the three soil types followed the order Pb > As > Hg. In soil S1, Pb concentrations showed a significant difference (*p* ≤ 0.05), with initial and final average values of 1997 ± 2 mg kg^−1^ and 1433 ± 46 mg kg^−1^, respectively. Overall, soils S1, S2, and S3 exhibited a slight decrease in metal concentrations, likely due to plant uptake, Hg volatilization, and/or Pb and As leaching towards the bottom of the containers in the experimental units.

It should be noted that the concentration of metals in the soil closest to the mine (S1) exceeded the maximum permissible limits established by the European Economic Community (1986) for Pb: 50–300 mg kg^−1^ and Hg: 1–1.5 mg kg^−1^, as well as the maximum permissible limit by the Eco SSL for As: 18 mg kg^−1^. In contrast, at S2 and S3, only Hg concentrations exceeded these permissible limits. Additionally, as shown in Fig. 2, metal concentrations decreased with increasing distance from gold mining activities.

Table 2 compares various studies that have reported high concentrations of Hg, Pb, and As in soils with mining and agricultural activities and in areas adjacent to mining sites. The Hg concentrations observed in soils S1, S2, and S3 in this study fall within the range typically found in mining areas at the national level, supporting previous findings from San Martín de Loba and Barranco de Loba (Bolívar). These soils originate from the same mining district, reinforcing the representativeness of the study.

**Table 2:** Report of concentrations of Hg, Pb and As in soils

Town	Soil use	Metal	Concentration (mg kg^−1^)	Reference
San Martín de Loba-Bolivar, Colombia	Miner	Hg	0.002–23.83	Rocha-Román et al., (2018)
Shaanxi, China	Miner		1.53–1054.97	Jia et al., (2018)
Miraflores-Quinchía, Colombia	Miner		7.1 ± 6.2	Camargo Garcia et al., (2015)
Barranco de Loba-Bolívar, Colombia	Miner		3.55–7.82	Pérez Vargas et al., (2015)
San Joaquín, México	Miner		2.4–4164	Martínez-Trinidad et al., (2013)
	Agricultural		0.5–314	
	Forestry		0.2–69.0	
Barranco de Loba-Bolívar, Colombia	Miner		6.91–3.13	Vidal Durango et al., (2010)
Cuenca del río Sinú, Colombia	Agricultural	Pb	0.071	Marrugo-Negrete et al., (2017)
Tongguan-Shaanxi, China	Miner		252–1295	Xiao et al., (2017)
Jilin, China	Miner		819.67	Chen et al., (2017)
Socha, Tasco, Samacá & Tenza, Boyaca, Colombia	Miner		0.03–4.06	Agudelo Calderón et al., (2016)
Abakaliki,Nigeria	Miner		1117	Oti Wilberforce and Nwabue (2013)
Paz de Río, Villavicencio-Meta, Colombia	Agricultural		14.52–15.11	Ramírez and Navarro (2015)
Villa de la Paz, México	Miner		458	Gamiño-Gutiérrez et al., (2013)
Mount Isa-Queensland, Australia	Miner		1560	Mackay et al., (2013)
Buriganga River, Bangladesh	Agricultural		30.70	Kabir et al. (2022)
Lago Nansi, China	Agricultural	As	6.3–13.6	Wang et al., (2019)
Salamanca, España	Miner		138–854	Abad-Valle et al., (2018)
Gral. Cerri & Alférez San Martín, Argentina	Agricultural		7.2–14.5	Blanco et al., (2018)
Tolima & Santander, Colombia	Sites near mining		14.8–41	Alonso et al., (2014)
Bosa, Cota & Ricaurte,Cundinamarca, Colombia	Agricultural		0.98–2.54	Barragan Moreno (2008)
La Mojana región, Colombia	Agricultural		2.18–18.89	Urango-Cárdenas et al., (2024)
Antioquia, Bolívar, Boyacá & Santander; Colombia	Miner		13.7–35.3	Alonso et al., (2014)
Buriganga River, Bangladesh	Agricultural		29.70	Kabir et al. (2022)
San Martín de Loba-Bolivar, Colombia	S1: Miner	Hg	22.13 ± 0.57	This study
		Pb	1997.02 ± 2.06	
		As	37.52 ± 0.48	
	S2: Sites near mining	Hg	5.38 ± 0.72	
		Pb	186.03 ± 3.93	
		As	15.70 ± 0.16	
	S3: Agricultural	Hg	2.05 ± 0.22	
		Pb	57.19 ± 5.72	
		As	7.90 ± 0.74	

In Mexico and China, mining and agricultural regions show Hg concentrations that are sometimes higher than those observed in Colombia. Additionally, Pb concentrations in all three soils in this study are higher than in some reference studies, while As concentrations are comparable to levels reported for various Colombian departments, including Bolívar. However, certain mining areas in Salamanca, Spain, display As concentrations exceeding those in S1, while soils in northern China report As levels similar to those found in S2 and S3.

These findings highlight that the highest concentrations of Hg, Pb, and As are typically found at mining sites. Mining activities—such as mineral processing, transport, and the release of tailings and wastewater—are major sources of metal contamination. Metals from mining areas can migrate into nearby soils through acid mine drainage or atmospheric deposition of windborne dust, depending on the local climate and hydrology (Díez et al., 2011; Esbrí et al., 2015). As a result, metals in mining zones often disperse into adjacent and agricultural soils, influenced by environmental factors such as rainfall and wind (Kwon et al., 2017).

On the other hand, chemical fractionation analysis allowed for determining the biological availability of metals (Zhang et al., 2018). Fractions 1 and 2 (F1 and F2) are considered bioavailable; Hg is associated with dissolved organic matter (without Hg-carbon bonds) or suspended mineral particles, but it does not appear as ionic species soluble in water (Pinedo-Hernández et al., 2015). Across the three soils studied, Hg showed low bioavailability in these fractions (< 1%). However, there remains a possibility of Hg bioaccumulation in plant tissues.

For As, the values are similar to those of Hg, with bioavailable percentages in fractions F1 and F2 remaining below 1% across all three soils. Among these fractions, metals exhibit higher bioavailability, with F1 being more accessible for biological use and more active under neutral pH conditions, while F2 is more susceptible to changes in pH (Zhang et al., 2017).

For Pb, the exchangeable fraction (F1) in the three soils had values ranging from over 2% to nearly 11% (S1: 5.34%, S2: 2.92%, and S3: 10.99%), while the carbonate fraction (F2) showed percentages between just over 1% and nearly 5% (S1: 1.31%, S2: 2.09%, and S3: 4.35%).

Regarding the bioavailable phases, studies have shown low bioavailability of Hg in mining and agricultural soils. For instance, 1.68% of Hg is available in Sihui soils (China) (J. Zhang et al., 2018), while a similar fraction (1%) was reported for agricultural soils in the Sinú river basin, Colombia (Marrugo-Negrete et al., 2017). Similarly, Pérez-Vargas et al. (2015) reported 4.1% and 4.3% for soils in the gold mining area of Barranco de Loba (Bolívar, Colombia). These values are higher than those found in this study. Additionally, Wang et al. (2014) found that bioavailable Hg in mining soils from Wanshan (China) represented only 0.01%, similar to the values obtained in this study.

For Pb, values around 3% were found in the bioavailable fractions in the agricultural soil S2, similar to those reported by Marrugo-Negrete et al. (2017). However, Kasemodel et al. (2016) found that some soils from a mining area in Adrianópolis, Brazil, contained 0.49% of Pb in the exchangeable fraction, which is lower than the values obtained in this investigation. They also reported 5.46% of Pb in the carbonate fraction, which is similar to the values found in soil S3. Li et al. (2015) found that in mining and smelting soils in China, the exchangeable fraction contained between 0.70% and 20.3% of Pb, and the carbonate fraction contained between 0.30% and 41% of Pb. The percentages obtained in this investigation for each soil fall within these ranges.

For As, our soils showed low bioavailability values, consistent with previous studies. Sofianska and Michailidis (2015) reported values of < 1% (0.78%) for As in the exchangeable fraction in agricultural soils of Drama plain (Macedonia, Greece). Barać et al. (2015) found similar values of < 1% (0.6%) for As in the bioavailable fraction in agricultural soils in the industrial and mining regions of northern Kosovo and southern Serbia. Anawar et al. (2006) reported As values between 0.006% and 0.47% in the bioavailable fraction. Additionally, Casado et al. (2007) found values of 0.14% and 0.38% for As in the first fraction in soils from a mining area in Spain.

Considering that the RAC is calculated based on the sum of percentages from F1 and F2, it can be concluded that there is no environmental risk for Hg and As across the three soils. For Pb, a low risk is observed in S1 and S2, while a medium risk is observed in S3. These findings align with other studies (Kasemodel et al., 2016; Marrugo-Negrete et al., 2017).

Thus, although the soils impacted by mining activities contain high concentrations of Hg, Pb, and As, the results of this study indicate that the bioavailability of these metals remains low. Consequently, the environmental risk posed is minimal to low.

### Morphometric and physiological variables of the species *C.annuum*

The morphometric variables—stem length, stem diameter, number of branches, and fruit count—for each evaluated soil are presented in Figure S1. Plants grown in S1 displayed significantly lower values (*p* ≤ 0.05) across all morphological variables, indicating phytotoxic effects. Specifically, plants in S1 had a stem length of 32 cm, a stem diameter of 5 mm, 14 branches, and no fruit, compared to plants grown in the other soils (Figure S1). In contrast, plants grown in S2 and S3 showed similar measurements, with stem lengths of 79 and 81.03 cm, stem diameters of 10.33 and 10 mm, 31 and 28 branches, and 7 and 11 fruits, respectively.

Given that *C.annuum* can reach up to 2 m in height (R. Bernal et al., 2012) and typically attains heights of 75–80 cm by 154 days of age (Sánchez et al., 2003), it can be concluded that plant growth in S2 and S3 was not impacted by soil conditions, as they achieved typical heights by 143 days.

The significant phytotoxic effects observed in plants grown in S1 may be attributed to the soil’s acid sulfate properties and high concentrations of Hg, Pb, and As. These metals are known to induce plant stress, leading to inhibited growth, structural damage, and reduced physiological and biochemical activities (Cheng, 2003; Gunes et al., 2009; A. Khan et al., 2015; Nagajyoti et al., 2010). Additionally, acid sulfate soils limit nutrient availability and hinder the absorption of essential bases, negatively impacting plant growth, development, and productivity. This reduces the sustainability and competitiveness of crops in such soils (Bernal et al., 2016; Montaño Santana & Forero Ulloa, 2013).

The leaf area for S1 was 89 ± 62 cm^2^, which was significantly smaller compared to S2 (640 ± 201 cm^2^) and S3 (360 ± 117 cm^2^) (*p* ≤ 0.05) (Figure S2a). On the other hand, the dry biomass was significantly higher (*p* ≤ 0.05) for plants that grew in S3 (root: 10.9 ± 1.4 g, stem: 15.1 ± 1.0 g, and leaf: 8.5 ± 0.3 g) compared to S1 (root: 0.98 ± 0.07 g, stem: 1.56 ± 0.16 g, and leaf: 1.43 ± 0.80 g) and S2 (root: 11.1 ± 0.7 g, stem: 10.7 ± 1.1 g, and leaf: 6.3 ± 0.2 g) (Figure S2b).

The smallest biomass was observed in the roots of S1, which were 11 times smaller than those in S2 and S3. Reduction in leaf area and biomass is a common effect in plants exposed to Hg, Pb, and As (Marrugo-Negrete et al., 2016a, 2016b; Rai et al., 2011; Rossato et al., 2012). This phenomenon can be attributed to plants allocating more energy to survival mechanisms due to the stress induced by these metals (Kumar & Aery, 2016). For instance, Szaková et al. (2007) reported leaf biomass reductions between 10 and 25%, and stem reductions of up to 10%, in pepper plants (*C.annuum*) grown under various As concentrations. Similarly, Irfan et al. (2010) found that tomato plants (*Solanum lycopersicum* L.) grown in soils with different Pb concentrations showed a reduction in dry biomass of roots and shoots compared to controls. Marrugo-Negrete et al. (2015) reported a maximum reduction of 16% in the dry biomass of Jatropha curcas plants grown in soils with varying Hg concentrations.

The contents of chlorophyll A, B, A/B ratio, A + B, and carotenoids did not differ significantly (p ≤ 0.05) among the plants grown in the three soils (Figure S3). These similar levels indicate that the plants were able to tolerate the metal-induced stress, as no signs of chlorosis or necrosis were observed. Similar findings have been reported in other studies, such as Rossato et al. (2012), where *Pluchea sagittalis* showed unaffected chlorophyll and carotenoid concentrations in the presence of Pb. Likewise, no significant differences were observed in chlorophyll and carotenoid contents in Capsicum species (*Capsicum chinense, C. annuum,* and *Capsicum frutescens*) under stress conditions (Okunlola et al., 2017).

In contrast, other studies have documented reductions in photosynthetic pigments due to metal exposure. For example, Gupta et al. (2013) found significant decreases in chlorophyll A, B, total chlorophyll, and carotenoids in *Pfaffia glomerata* exposed to Hg, As, and Pb. Similarly, Trentin et al. (2025) reported reduced chlorophyll A and B in plants exposed to Hg and Pb, though they observed no significant changes in the A/B ratio. Irfan et al. (2010) noted a significant decrease in chlorophyll A, B, and A + B in *S. lycopersicum L.* under high Pb concentrations, with no significant differences in the A/B ratio. Additionally, Stoeva et al. (2005) found a reduction of 22% in chlorophyll and 19% in carotenoids in *Phaseolus vulgaris L.* exposed to high As concentrations.

In general, *C. annuum* plants grown in S1 showed the greatest developmental impact. Conversely, plants in S2 and S3 did not exhibit significant effects, similar to the findings of Sierra et al. (2008) for eggplant (*Solanum melongena*), which demonstrated normal development in soils from a mining district with concentrations of 14.16 ± 0.65 mg Hg kg^−1^.

### Concentration of metals in plant tissues

**Fig. 3:**
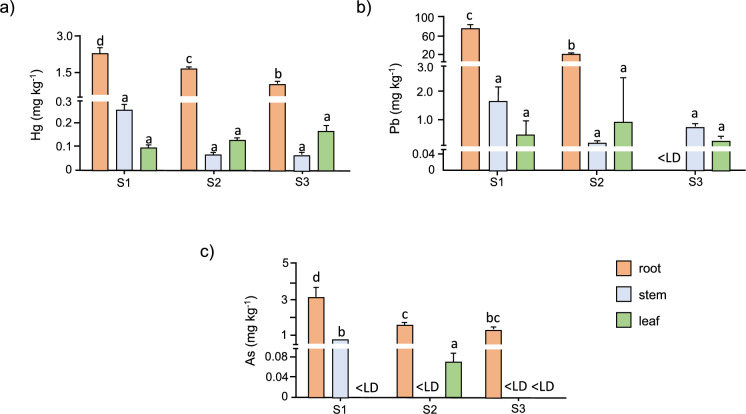
Hg, Pb, and As concentrations in plant tissues (roots, stems, and leaves). **a** Concentration of Hg in root, stem and leaves. **b** Pb concentration in root, stem and leaves. **c** As concentration in root, stem and leaves. < LD: less than the detection limit

The concentrations of Hg, Pb and As in roots, stems and leaves in the three evaluated soils are shown in Fig. 3.

The mean comparison test for metal accumulation in plant tissues revealed significant differences across the three soils: Hg in the root, Pb in the root for S1 and S2, and As in the root and stem for S2, compared to other plant tissues. Generally, metal accumulation was higher in roots than in leaves and stems.

For Hg accumulation (Fig. 3a), the order in S1 was root > stem > leaves, while in S2 and S3 it was root > leaves > stem. The highest Hg accumulation was observed in roots across all soils, with values of 2.29 mg kg^−1^ in S1, 1.61 mg kg^−1^ in S2, and 0.95 mg kg^−1^ in S3, consistent with high Hg concentrations in roots reported by Pérez-Vargas et al. (2015) for *C. annuum*. The authors explain that Hg binds strongly to cell walls in the root due to its direct contact with contaminated soil. Similar to S2 and S3, Marrugo-Negrete et al., (2016a, 2016b) observed that Hg accumulated most in the roots, followed by leaves and stem in *C. annuum*, attributing lower stem concentrations to its role in nutrient transport via xylem and phloem, facilitating Hg transport to aerial parts. Additionally, Hg can accumulate in leaves through air exposure, as Hg is volatile.

For Pb accumulation (Fig. 3b), levels were much higher than for the other metals, influenced by high Pb concentrations and bioavailability in the soils. Roots, being in direct contact with soil, accumulated high Pb concentrations in S1 and S2, with values of 70.6 mg kg^−1^ and 28.3 mg kg^−1^, respectively, while plants in S3 showed lower Pb accumulation. The Pb accumulation order was S1: root > stem > leaves, S2: root > leaves > stem, and S3: stem > leaves > root. In many plants, Pb content generally decreases in the order root > leaves > stem, as seen in S2 (Feleafel & Mirdad, 2013). However, variations in accumulation order, as observed in S1 and S3, are common. Antonious (2016) reported the order leaves > root > stem for Pb in *C. annuum* (hot pepper), with leaf and stem values similar to those in S2. For hot pepper (*C. annuum L.*), Hong et al. (2008) reported leaf and root Pb values comparable to those found in S3. Studies such as Li et al. (2018) found high Pb concentrations in Oryza sativa L., showing an accumulation order similar to S1.

For As accumulation (Fig. 3c), the order was S1: root > stem > leaves, S2: root > leaves > stems, and S3: root > stem = leaves. The root showed higher As concentrations than stem and leaves, likely because it is the primary contact point with the metal. As reported by Moreno-Jiménez et al. (2012), most plants retain As in their roots, where it enters the root apoplast through diffusion and moves into plant cells via the symplast. Through reduction in the root, plants may restrict As flow to aerial tissues, explaining the low As concentrations in stems and leaves. Similar to S1, Száková et al. (2007) reported As accumulation in *C. annuum* tissues in the order root > stem > leaves, with stem values comparable to those in S1.

### Bioconcentration and translocation factors

Both BCF and TF factors were calculated to assess the ability of plants to accumulate metals in their roots from the soil and transfer them to aerial tissues relative to the root concentration. For *C. annum* plants, both BCF and TF values were less than 1 for all metals tested (Table 3), indicating that metals were transferred to the plant but not substantially, thus classifying this species as a non-accumulator (Ruiz Huerta & Armienta Hernández, 2012). Notably, the highest values were observed for Hg and for Pb in site S3, although Pb was not calculated for the root due to concentrations below the detection limit. Additionally, there was a general trend of increased BCF values as metal concentrations decreased in the soil.

**Table 3:** FBC and FT values of C. *annuum* for Hg, Pb and As

Soil	BCF			TF		
	Hg	Pb	As	Hg	Pb	As
S1	0.10	0.04	0.09	0.04	0.01	0.01
S2	0.30	0.15	0.10	0.07	0.04	0.04
S3	0.47	0.00	0.16	0.15	NC	0.02

NC: value not calculated

In related research, Pérez Vargas et al. (2015) reported for *C. annum* a BCF of 0.83 and a TF of 1.19 for Hg, higher than our findings. However, the Hg concentrations in their study ranged from 0.23–6.32 mg kg^−1^ in soil, suggesting that BCF increases at lower metal concentrations, as seen here where S3 exhibited a higher BCF despite its lower soil Hg levels compared to sites S1 and S2, where soil concentrations were higher.

Other studies further support these findings. Barać et al. (2015) reported an TF for Pb < 1 (0.007) in *Solanum tuberosum* L., while Khan et al. (2017) reported a BCF for Pb < 1 (0.17–0.20) in the same species. Similarly, Yu et al. (2012) observed BCF and TF values < 1 for Hg, Pb, and As in *Brassica napus* L., except for Hg. Wang et al. (2019) found that BCF values for *Oryza sativa* L. ranged from 0.22 to 0.89 and TF values from 0.13 to 1.52 for As, depending on the genetic variety and metalloid availability in the soil. These studies collectively illustrate that metal absorption and translocation vary across plant species and are influenced by factors such as metal content and bioavailability, soil pH, and organic matter, as well as plants’ cellular mechanisms for metal tolerance (Kumar & Aery, 2016; Mitra, 2015; Zheng et al., 2018).

### Concentrations of metals in the fruits of *C. annuum*

The concentrations of Hg, Pb, and As in four harvests of *C.annuum* from sites S2 and S3, where production occurred, are presented in Table 4. In S2, Hg concentrations in fruits ranged from 0.05 ± 0.01 to 0.100 ± 0.003 mg kg^−1^, whereas in S3, the concentration was as low as 10^–4^ mg kg^−1^. These levels are below the 0.13 mg kg^−1^ reported by Pérez-Vargas et al. (2015) for the same species grown in mining soils in South Bolívar, Colombia. Similarly, Zheng et al. (2007) found Hg concentrations in *C. annuum L.* var. *grossum* L. ranging from 0.006 to 0.095 mg kg^−1^, which aligns with the values obtained in our study.

**Table 4:** Concentrations of metals (in mg kg^−1^) in the fruit of *C. annuum*

Crop	Soil	Hg	Pb	As
C1	S2	0.07 ± 0.04	0.090 ± 0.004	< 0.05
C2	S2	0.05 ± 0.01	< 0.08	< 0.05
C3	S2	0.100 ± 0.003	< 0.08	< 0.05
C4	S2	0.05 ± 0.01	< 0.08	< 0.05
C1	S3	1 × 10^–4^	< 0.08	< 0.05
C2	S3	1 × 10^–4^	< 0.08	< 0.05
C3	S3	1 × 10^–4^	< 0.08	< 0.05
C4	S3	4 × 10^–4^	< 0.08	< 0.05
	MAC	0.01^a^	0.1^a^	0.5^a^
		–	0.05^b^	–

MAC: Maximum Concentration allowed in food (Vegetables). ^a^GB 2762–2022 ^b^Codex Alimentarius (CXS 193–1995, amended 2024)

For Pb and As, concentrations in all harvests were generally below detection limits (< 0.08 mg kg^−1^ for Pb and < 0.05 mg kg^−1^ for As), except for the first harvest in S2, where Pb reached an average concentration of 0.090 ± 0.004 mg kg^−1^. Previous studies have reported relatively low Pb and As levels in various *C.annuum* fruits. For instance, Zhou et al. (2016) found Pb and As concentrations of 0.560 ± 0.009 and 0.016 ± 0.005 mg kg^−1^, respectively, in red pepper (*C. annuum L.*) grown near a mining area. Islam and Hoque (2014) reported Pb and As concentrations of 0.17 ± 0.04 and 0.01 ± 0.00 mg kg^−1^, respectively, in *C. annuum* cultivated in an industrial area, while Rodríguez-Iruretagoinea et al. (2015) documented levels of 0.134 mg kg^−1^ for Pb and 0.125 mg kg^−1^ for As in peppers.

In greenhouse-grown hot peppers, Antonious et al. (2016) recorded Pb levels of 0.2743 ± 0.018 mg kg^−1^. Bundschuh et al. (2012) reported a significantly higher As concentration (6.3 ± 1.4 mg kg^−1^) for peppers grown in mining areas, while Gebrekidan et al. (2013) found elevated Pb concentrations of 2.07 ± 0.67 and 2.16 ± 0.34 mg kg^−1^ in green peppers from two industrially contaminated sites. Notably, most of these studies indicate a trend of higher Pb concentrations compared to As, a trend also evident in the first harvest of S2 in this study.

Hg concentrations in fruits exceeded the maximum allowed concentration (MAC) of 0.01 mg kg^−1^ established by the National Health Commission of China (GB 2762-2022, 2022) for vegetables in all harvests from S2. This is concerning given mercury’s well-documented toxicological effects, including neurotoxicity and renal impairment, and its ability to bioaccumulate in human tissues with repeated dietary exposure (De la Ossa et al., 2023; WHO, 2024).

For Pb, the concentrations observed in the first harvest from S2 did not exceed the 0.1 mg kg^−1^ limit established by the Chinese National Food Safety Standard (GB 2762-2022), but were higher than the more stringent 0.05 mg kg^−1^ threshold established by the Codex Alimentarius (CXS 193-1995, 1995, amendment 2024). This finding is relevant because Pb is a highly toxic element that can impair neurological development, cardiovascular function, and renal health even at low exposure levels. Children are particularly susceptible to its effects (Gundacker et al., 2021; Rossato et al., 2012).

In contrast, As concentrations in all harvests remained below the MAC of 0.5 mg kg^−1^ (GB 2762-2022). However, As is classified as a human carcinogen, and its persistence in the edible tissues of crops cultivated in contaminated soils requires careful monitoring, especially in cases of long-term exposure (Urango-Cárdenas et al., 2024). Overall, these results suggest that although fruits from S3 contained negligible levels of metals, those from S2 exhibited Hg and Pb concentrations at or above internationally recognized thresholds. This raises concerns about potential chronic health risks for consumers in mining-impacted areas (Fig. 3).

Conversely, in soil S1, which exhibited the highest concentrations of Hg, Pb, and As, no fruit production was documented. This decline in reproductive success can be attributed to the combined impact of direct metal toxicity on plants and indirect effects mediated by soil degradation. Elevated levels of heavy metals have been demonstrated to suppress microbial biomass and diversity, inhibit key soil enzymes (phosphatase, urease, catalase), and disrupt nutrient cycling, thereby reducing soil fertility and nutrient availability (Angon et al., 2024). At the physiological level, exposure to critical concentrations of Pb, Hg, and As has been demonstrated to induce oxidative stress and the generation of reactive oxygen species (ROS). This, in turn, has been shown to decrease chlorophyll synthesis, disrupt photosynthesis, and interfere with nutrient uptake (Ca, Fe, Zn). The net effect of these changes is an ultimate compromise of the vegetative-to-reproductive transition (Angon et al., 2024; Bortoloti & Baron, 2022; Marrugo-Negrete et al., 2015). In such conditions, plants prioritize stress defense and maintenance over reproduction, thereby explaining the absence of flowering and fruit set in S1 (Angon et al., 2024).

### Health risk assessment

#### Estimated daily intake for metals

The EDI of Hg from fruits cultivated in treatments S2 and S3 was 0.037 and 0.00006 µg kg^−1^ BW day^−1^, corresponding to weekly intakes of 0.26 and 0.00042 µg kg^−1^ BW, respectively. These values are low compared to the provisional tolerable weekly intake (PTWI) for Hg (4 µg kg^−1^ BW) established by JECFA (2011), indicating a low risk from Hg exposure under the studied conditions. For Pb, the intake is 0.046 µg kg^−1^ BW day^−1^) for fruits from S2. As intake was not calculated as concentrations were below the detection limit.

For Pb, risk assessment is based on benchmark dose modeling. EFSA (2010) established a BMDL01 of 0.5 µg kg^−1^ BW day^−1^ associated with a one-point reduction in IQ in children, while for adults, benchmark values of 0.63 µg kg^−1^ BW day^−1^ for chronic kidney disease (CKD) and 1.50 µg kg^−1^ BW day^−1^ for systolic blood pressure (SBP) were identified. These benchmarks were specifically derived for lead and form the basis for calculating the margin of exposure (MOE). In this study, MOE values were 33 for adults and 11 for children, both above the EFSA threshold of concern (MOE ≥ 10). However, the value for children lies close to the lower boundary, reflecting their higher vulnerability to Pb neurotoxicity and suggesting that, although the immediate risk is low, chronic exposure may still represent a concern for sensitive populations (EFSA, 2010; JECFA, 2011).

These findings are consistent with those reported by Pérez-Vargas et al. (2015) and Shaheen et al. (2016), indicating that the consumption of fruits from these plants does not pose an immediate health risk to local consumers. However, given the bioaccumulative nature of Pb, Hg, and As, and considering that the calculated MOE for Pb in children (11) is close to the lower threshold of concern, continuous exposure may still pose long-term health risks, particularly for vulnerable populations.

#### Non-carcinogenic and carcinogenic risk indices

Table 5 presents the values of THQ for Hg and Pb, HI, and ILCR obtained for *C. annuum* cultivated in soils S2 and S3. Cases marked as “NC” correspond to concentrations below the detection limit of the analytical method, for which risk values could not be calculated.

**Table 5:** Non-carcinogenic (THQ, HI) and carcinogenic (ILCR) risk indices for *C.annuum* cultivated in soils S2 and S3

Crop	Soil	THQ		HI	ILCR
		Hg	Pb		
C1	S2	0.119	0.0131	0.1321	3.90E−04
C2	S2	0.119	NC	0.119	NC
C3	S2	0.17	NC	0.17	NC
C4	S2	0.085	NC	0.085	NC
C1	S3	1.70E−04	NC	0.00017	NC
C2	S3	1.70E−04	NC	0.00017	NC
C3	S3	1.70E−04	NC	0.00017	NC
C4	S3	6.80E−04	NC	0.00068	NC

NC: Value not calculated

In crops grown in soils from site S2, HQ values for Hg ranged between 0.085 and 0.17, with a single case of Pb detected (0.0131 in C1-S2), whereas HI, remained below 1 in all cases, indicating no significant non-carcinogenic risk from fruit consumption under these conditions. However, the incremental lifetime cancer risk (ILCR) value of 3.90 × 10^–4^ recorded in C1-S2, associated with Pb exposure, falls within the range indicative of potential carcinogenic risk, though still at a low-to-moderate level according to USEPA benchmarks.

In contrast, crops cultivated in soils from site S3 exhibited markedly lower HQ values for Hg (on the order of 10^−4^ to 10^−3^). No Pb values or ILCR were calculated for this site, as concentrations were below the detection limit of the analytical method. Consequently, THQ values were also extremely low (< 0.001), effectively ruling out non-carcinogenic risks in these samples.

Overall, fruits cultivated in S3 present negligible exposure to the evaluated metals, whereas in S2, measurable HQ values and an ILCR associated with Pb highlight the importance of considering bioaccumulation and the potential implications of chronic exposure, particularly for vulnerable populations. The absence of calculations (NC) in most cases confirms that Pb and As concentrations were below detection limits, thereby reducing immediate concern for public health risks.

These results highlight the need to assess risk within broader management practices. This is essential to safeguard food security for local populations in agricultural-mining regions of Colombia. These regions experience significant contamination from gold mining. To achieve this objective, strategies must align with the principles of the circular economy and nature-based solutions. These solutions must address pollution in addition to enhancing soil productivity and community resilience. Field trials in Ayapel (Córdoba) have shown that using native plants like *Jatropha curcas, Piper marginatum* and *Cecropia peltata* can restore vegetation and reduce soil Hg concentrations by nearly 30% (Obaji Bernal et al., 2025; Vidal Durango et al., 2010). Similarly, using biochar, vermicompost, and pumice stone to improve soil quality has achieved retention efficiencies above 90% for multiple metals. These approaches, already tested in mining regions in Colombia, show how to assess risks and restore resources. Our results emphasize the need to protect food security and ensure sustainable agricultural development in mining communities.

## Conclusions

The study findings suggest that *C.annuum* can tolerate the stress associated with mining soils, which are characterized by high metal concentrations and acidic sulfate conditions, as evidenced by its stable growth in treatments S2 and S3. In general, plants in these treatments showed higher concentrations of Hg, Pb, and As in roots compared to other tissues, as the roots are in direct contact with soil metals. The BCF and TF values indicate that this species does not act as an accumulator for the metals studied.

Fruits harvested from S2 and S3 contained Hg levels exceeding the MAC, while Pb and As concentrations were mostly below the detection limit, posing minimal risk. Additionally, weekly intake values for each metal were below the toxicological reference levels of the Codex Alimentarius (CXS 193-1995, amended 2024), suggesting that consuming *C. annuum* fruits grown in similar soil conditions does not present a health risk.

Despite the elevated concentrations of Hg, Pb, and As in soils from the municipality of San Martín de Loba (Bolívar Department), *C. annuum* showed limited translocation of these elements to edible tissues, likely due to low metal bioavailability and intrinsic plant exclusion mechanisms. These findings suggest that, in gold mining regions where agriculture and small household orchards coexist, the cultivation of non-accumulator vegetable species may reduce direct dietary exposure, particularly when crops are grown at moderate distances from mining activities.

In the local context of gold mining regions, priority mitigation strategies should include: (i) the establishment of buffer zones between mining operations and agricultural fields, particularly within the first kilometer from active mining sites; (ii) the preferential cultivation of low-translocation or non-edible plant species in highly contaminated soils to reduce direct human exposure; (iii) the implementation of phytostabilization using non-edible native species to reduce metal dispersion and prevent wind erosion of mine tailings; (iv) the application of organic soil amendments to decrease Hg and Pb bioavailability; (v) regular, site-specific and community-supported monitoring programs targeting toxic metals in soils, irrigation water, and harvested fruits; and (vi) the exclusive use of verified contaminant-free water sources for irrigation to prevent secondary metal inputs. In addition, post-harvest practices, such as thorough washing, peeling when applicable, and controlled handling during storage and transport, should be promoted to further minimize dietary exposure.

These results provide a scientifically sound basis for evidence-based public policies aimed at protecting food safety and human health in mining-influenced agricultural systems. Such policies should integrate geochemical risk mapping, crop selection guidelines, and farmer training programs focused on contamination management in small-scale agricultural environments typical of gold mining regions in Colombia and other countries.

Nevertheless, it must be acknowledged that this study was conducted under controlled screen-house conditions, which do not fully capture field-scale processes such as seasonal climatic variability, rainfall-driven leaching, or irrigation practices. Furthermore, key factors influencing metal mobility—such as soil microbial activity and Fe content, known to regulate As and trace metal behavior—were not evaluated. Addressing these limitations through long-term field studies should be a priority for future research to refine risk management strategies under real agricultural conditions.

## Supplementary Information

Below is the link to the electronic supplementary material.

Supplementary file1 (DOCX 150 KB)

## Data Availability

No datasets were generated or analysed during the current study.
