# Supplementary material for: Metal uptake in sweet peppers cultivated in soils contaminated by artisanal gold mining: implications for food safety
Source: Environ Geochem Health. 2026 Mar 9;48(5):223. doi: 10.1007/s10653-026-03077-z (PMC12971792; doi:10.1007/s10653-026-03077-z)

**SUPPLEMENTARY MATERIAL FOR PAPER**

**Metal uptake in sweet peppers cultivated in soils contaminated by artisanal gold mining: implications for food safety**

Elvia Valeria Durante-Yánez^1^, Iván David Urango-Cárdenas^1^, Germán Holland Enamorado-Montes^1^, Marisol Laza-Durante^1^, Enrique Combatt Caballero^2^, José Luis Marrugo-Negrete^1*^, Roberth Paternina-Uribe^1^, Sergi Díez^3,*^

^1^Universidad de Córdoba, Faculty of Basic Sciences, Department of Chemistry, Water Research Group, Applied Chemistry and Environmental, Monteria, Colombia.

^2^Universidad de Córdoba, Faculty of Engineering, Monteria, Colombia.

^3^Environmental Chemistry Department, Institute of Environmental Assessment and Water Research, IDAEA-CSIC, E-08034, Barcelona, Spain

**Figure S1.** Morphometric variables at the end of the test. a) Length of the stem. b) Diameter of the stem. c) Number of the leaf. d) Number of fruits.


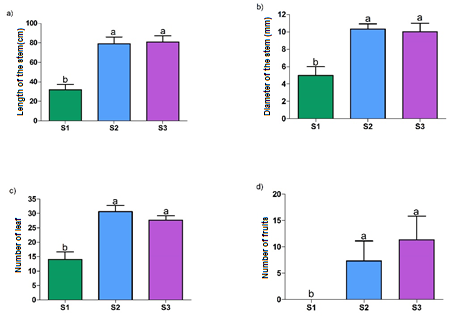


**Figure S2.** a) Leaf area for three soils. b) Dry biomass (root, stalk and soil) for the three soils.


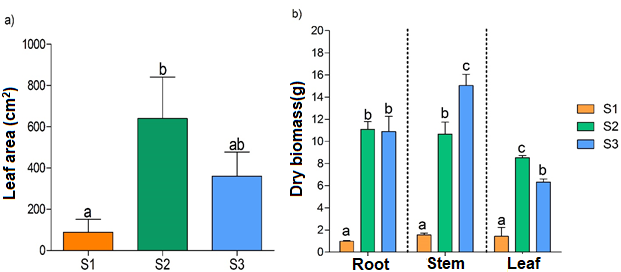


**Figure S3.** Chlorophyll A, B, A/B, A+B and carotenoids for the three soils.


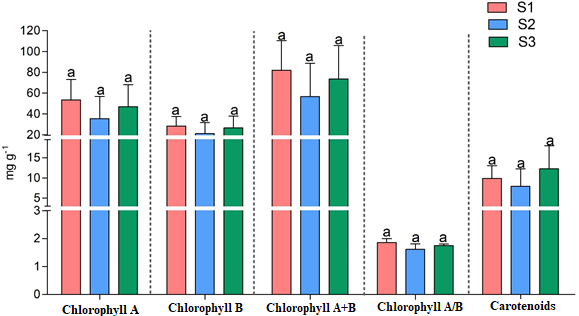

Supplement: Supplementary file 1 — Supplementary file1 (DOCX 150 KB) [file 10653_2026_3077_MOESM1_ESM.docx]
